# Nasal high flow higher than 60 L/min in patients with acute hypoxemic respiratory failure: a physiological study

**DOI:** 10.1186/s13054-020-03344-0

**Published:** 2020-11-23

**Authors:** Maria Cristina Basile, Tommaso Mauri, Elena Spinelli, Francesca Dalla Corte, Giacomo Montanari, Ines Marongiu, Savino Spadaro, Alessandro Galazzi, Giacomo Grasselli, Antonio Pesenti

**Affiliations:** 1 Department of Anesthesia, Critical Care and Emergency, Fondazione IRCCS Ca’ Granda Ospedale Maggiore Policlinico, Milan, Italy; 2 Department of Pathophysiology and Transplantation, University of Milan, Via F. Sforza 35, 20122 Milan, Italy; 3 Intensive Care Unit, Department of Morphology, Surgery and Experimental Medicine, Sant’Anna University Hospital, Ferrara, Italy; 4 Direction of Healthcare Professions, Fondazione IRCCS Ca’ Granda Ospedale Maggiore Policlinico, Milan, Italy

**Keywords:** Acute hypoxemic respiratory failure, Nasal high flow, Patient self-inflicted lung injury, Comfort

## Abstract

**Background:**

Nasal high flow delivered at flow rates higher than 60 L/min in patients with acute hypoxemic respiratory failure might be associated with improved physiological effects. However, poor comfort might limit feasibility of its clinical use.

**Methods:**

We performed a prospective randomized cross-over physiological study on 12 ICU patients with acute hypoxemic respiratory failure. Patients underwent three steps at the following gas flow: 0.5 L/kg PBW/min, 1 L/kg PBW/min, and 1.5 L/kg PBW/min in random order for 20 min. Temperature and FiO_2_ remained unchanged. Toward the end of each phase, we collected arterial blood gases, lung volumes, and regional distribution of ventilation assessed by electrical impedance tomography (EIT), and comfort.

**Results:**

In five patients, the etiology was pulmonary; infective disease characterized seven patients; median PaO_2_/FiO_2_ at enrollment was 213 [IQR 136–232]. The range of flow rate during NHF 1.5 was 75–120 L/min. PaO_2_/FiO_2_ increased with flow, albeit non significantly (*p* = 0.064), PaCO_2_ and arterial pH remained stable (*p* = 0.108 and *p* = 0.105). Respiratory rate decreased at higher flow rates (*p* = 0.014). Inhomogeneity of ventilation decreased significantly at higher flows (*p* = 0.004) and lung volume at end-expiration significantly increased (*p* = 0.007), but mostly in the non-dependent regions. Comfort was significantly poorer during the step performed at the highest flow (*p* < 0.001).

**Conclusions:**

NHF delivered at rates higher than 60 L/min in critically ill patients with acute hypoxemic respiratory failure is associated with reduced respiratory rate, increased lung homogeneity, and additional positive pressure effect, but also with worse comfort.

## Background

Non-intubated patients with acute hypoxemic respiratory failure (AHRF) are characterized by derangements of gas exchange and respiratory mechanics [1, 2]. These contribute to an increase in the respiratory drive which, in turn, may lead to excessive effort, the main determinant of patient self-inflicted lung injury (P-SILI) and diaphragm myotrauma [3, 4]. First-line approach by noninvasive respiratory support should aim to preserve physiological spontaneous breathing, which is associated with multiple beneficial effects, by granting improved gas exchange and mechanics [5, 6]. Ineffective noninvasive support can lead to delayed intubation and poor clinical outcomes [7].

Nasal high flow (NHF) delivers heated and humidified air/oxygen mixture through specific prongs [8]. Previous studies showed both the physiological and clinical benefits of noninvasive support by NHF in AHRF patients [9, 10], to the point that NHF can already be considered as the recommended first-line noninvasive approach [11–13]. However, NHF fails to avoid intubation in around 30–40% of AHRF patients and research in this field should be aimed at finding more effective strategies able to decrease failure rate [14].

Previous studies showed that the physiological effects of NHF are correlated with the set flow rate [15]. Oxygenation, positive end-expiratory pressure (PEEP) effect, inspiratory effort, and CO_2_ clearance improved at higher flows, albeit with some degree of variability. In clinical practice and published studies, the flow range for NHF is up to 60 L/min, and only one study in healthy volunteers explored the physiological effects of NHF at flow rate higher than 60 L/min, describing higher PEEP effect and decreased respiratory rate [16]. In the present study, we reasoned that NHF delivered at flow rates higher than 60 L/min in AHRF patients might be associated with positive physiological effects improving lung protection and potentially decreasing the risk of failure in comparison to current clinically used flow rates. We also assessed comfort at these very high flow rates, as patient tolerance is a key factor for the clinical success of NHF.

## Methods

### Study population

We enrolled 12 non-intubated acute hypoxemic respiratory failure patients admitted to the intensive care unit (ICU) of the Fondazione IRCCS Ca’ Granda Ospedale Maggiore Policlinico, Milan, Italy. Inclusion criteria were: new or worsening respiratory symptoms (e.g., dyspnea, shortness of breathing) following a known clinical insult (e.g., pneumonia) lasting < 1 week; arterial partial pressure of oxygen/fraction of inspired oxygen (PaO_2_/FiO_2_) ≤ 300 while receiving additional oxygen as per clinical decision; evidence of pulmonary infiltrates on chest X-ray. Exclusion criteria were: age < 18 years; presence of tracheostomy; hemodynamic instability (hypotension with mean arterial pressure < 60 mmHg despite volume loads or vasoactive drugs); evidence of pneumothorax on chest X-ray or computed tomography scan; respiratory failure explained by cardiac failure or fluid overload; severe chronic obstructive pulmonary disease; history of nasal trauma and/or deviated nasal septum; altered mental status; contra-indication to electrical impedance tomography (EIT) monitoring (e.g., patient with implantable defibrillator); impossibility to position the EIT belt (e.g., wound dressings or chest drains). The Ethical Committee of the Fondazione IRCCS Ca’ Granda Ospedale Maggiore Policlinico, Milan, Italy, approved the study (reference number: 665_2018), and informed consent was obtained from each patient.

### Data collection

At enrolment, the following variables were collected: sex, age, body mass index (BMI), predicted body weight (PBW), Sepsis-related Organ Failure Assessment (SOFA) score, Simplified Acute Physiology Score (SAPS) II, and PaO_2_/FiO_2_ at ICU admission, etiology, and number of quadrants involved on chest X-ray.

### EIT monitoring

An EIT-dedicated belt containing 16 equally spaced electrodes was placed around each patient’s thorax at the fifth or sixth intercostal space and connected to a commercial EIT monitor (PulmoVista 500; Dräger Medical GmbH, Lübeck, Germany). During the study, EIT data were generated by applying small alternate electrical currents rotating around the patient’s thorax at 20 Hz, so that tomographic data were acquired every 50 ms throughout all study phases and stored for offline analyses performed by dedicated software (Dräger EIT Data Analysis Tool and EITdiag; Dräger Medical GmbH, Lübeck, Germany). In one patient, EIT data could not be analyzed because of poor quality of the recorded tracings.

### Study protocol

Patients were kept in the semi-recumbent position without sedation. A calm environment was ensured around the patients throughout the study. Each patient underwent three study phases in computer-generated random order, with each phase lasting 20 min:

- NHF with gas flow set at 0.5 L/kg PBW/min (NHF-0.5)
- NHF with gas flow set at 1.0 L/kg PBW/min (NHF-1)
- NHF with gas flow set at 1.5 L/kg PBW/min (NHF-1.5)

In case of severe discomfort (none for NHF-0.5, n = 1 for NHF-1 and n = 2 for NHF-1.5), flow was reduced by 5 L/min step until patient tolerance improved. The NHF apparatus was custom-made by two parallel air/oxygen blenders and two parallel active heated humidifiers connected to a y-piece and to a single nasal cannula, as previously described [16]. The system can deliver fully humidified gas flows between 4 and 120 L/min at FiO_2_ between 0.21 and 1.0. NHF was delivered through specific nasal prongs of medium or large size (Fisher and Paykel Healthcare, Auckland, New Zealand) to fit the size of the nares. The set FiO_2_ was chosen to target a peripheral oxygen saturation of 90–96% during the first step and was kept constant during all phases. Patients did not receive any instruction on mouth opening or closing.

### Target physiological variables

Toward the end of each study phase, we collected peripheral oxygen saturation, arterial blood gas analysis, respiratory rate (RR) and hemodynamics. Additionally, comfort score was reported by the patient through visual numerical scale (VNS) ranging between 0 (extreme discomfort) and 10 (very comfortable).

### EIT variables

The raw EIT data recorded during the last minutes of each step were analyzed offline. We divided the EIT lung-imaging field into two regions of interest: From halfway down, we identified the dependent dorsal lung region, while the other half represented the non-dependent ventral region. We measured the following EIT parameters:

1. Corrected minute ventilation (MV), measured as the minute ventilation expressed in arbitrary units multiplied by the ratio of the patient’s PaCO_2_ during each phase divided by 40 mmHg, with lower values indicating enhanced CO_2_ clearance, less CO_2_ production, or both;
2. Global and regional changes in end-expiratory lung impedance (corresponding to changes in end-expiratory lung volume) expressed in arbitrary units of impedance change from the baseline NHF-0.5 step (∆EELI, ∆EELI_non-dep_, and ∆EELI_dep_, respectively);
3. Global Inhomogeneity (GI) Index, as previously described [17]. Higher GI values indicate more inhomogeneous distribution of ventilation.

### Statistical analysis

Sample size was similar to previous studies [4, 17, 18]. Normally distributed variables are expressed as mean ± standard deviation, while median and interquartile range (IQR) were used to report non-normally distributed variables. Differences between variables across study phases were tested by one-way analysis of variance (ANOVA) for repeated measures or by one-way repeated measure ANOVA on ranks, as appropriate. Post hoc correction for all pair-wise multiple comparison procedures was performed using Bonferroni or Dunn’s method for non-parametric variables. A level of *p* < 0.05 was considered to be statistically significant. Statistical analyses were performed with SigmaPlot 11.0 (Systat Software Inc., San Jose, CA).

## Results

### Study population

We enrolled 12 patients, 4 (33%) women, with a median age of 70 (IQR 62–80) years. Patients presented a SAPS II score at ICU admission of 36 (22–44) and a SOFA score on the day of the study of 4 (3–7). Five patients (42%) had pulmonary etiology of AHRF and 9 (75%) had bilateral infiltrates on chest X-ray. Median PaO_2_/FiO_2_ at ICU admission was 213 (136–232). Main characteristics of the study population are shown in Table 1. None of the patients received sedative drugs during the study.

**Table 1:** Main characteristics of the study population

Patient	Sex	Age (year)	BMI (kg/m^2^)	SOFA score	SAPS II score at ICU admissison	PaO_2_/FiO_2_	Etiology of AHRF	Days since diagnosis of AHRF (no.)	Number of chest X-ray quadrants involved (no.)
1	F	85	21	6	33	273	Trauma	3	2
2	F	78	21	15	52	236	Pneumonia	1	1
3	M	82	29	9	42	229	Septic Shock	1	1
4	F	69	29	4	40	148	Pneumonia	1	2
5	M	70	24	5	46	221	Pneumonia	1	2
6	F	77	25	9	55	107	Septic Shock	1	1
7	M	52	26	3	20	106	Pneumonia	1	3
8	M	56	31	4	23	236	Trauma	5	2
9	M	40	24	3	22	206	Pneumonia	1	2
10	M	71	23	3	27	196	Postoperative	0	2
11	M	69	31	4	18	124	Postoperative	1	2
12	M	84	25	6	43	223	Septic Shock	1	4
Median (IQR)	8 M 4 F	70 (62–80)	25 (23–29)	4 (3–7)	36 (22–44)	213 (136–232)	Pulmonary: 5 Extra-pulmonary: 7 Infective: 7 Non-infective: 5	1 (1–1)	2 (2–2)

M, male; F, female; BMI, Body Mass Index; SOFA, sequential organ failure assessment; SAPS, simplified acute physiology score; AHRF, acute hypoxemic respiratory failure; PaO_2_/FiO_2_, arterial partial pressure of O_2_/inspired fraction of O_2_ratio; ICU, intensive care unit

### Effects of increasing set flow rate on target physiological variables

**Fig. 1:**
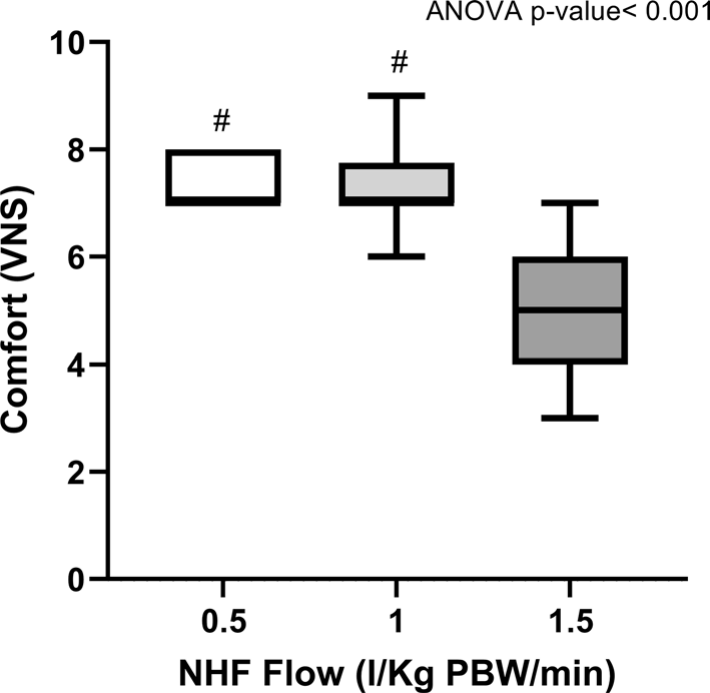
Patient comfort by visual numerical scale during NHF steps. Comfort during NHF-1.5 was significantly lower compared to NHF-1 and NHF-0.5. Post hoc correction was performed using Bonferroni test (#*p* < 0.05 vs. NHF-1.5)

The set flow rate during each step progressively increased from NHF-0.5 to NHF-1.5 (*p* < 0.001) (Table 2): the range of flow rate during HNF 1.5 was 75–120 L/min. PaO_2_/FiO_2_ and peripheral oxygen saturation increased with flow, albeit nonsignificantly (*p* = 0.064 and *p* = 0.139, respectively) (Table 2). PaCO_2_ remained stable (*p* = 0.108), as well as arterial pH (*p* = 0.105); the respiratory rate needed to obtain stable gas exchange decreased at higher flow rates (*p* = 0.014) but corrected minute ventilation remained stable (*p* = 0.068) (Table 2). Changes in flow had no clinically significant effect on vital signs such as mean arterial pressure (*p* = 0.447) and heart rate (*p* = 0.391) (Table 2). Patient self-reported comfort was significantly higher during steps performed at lower flows (7 ± 1 during both NHF-0.5 and NHF-1), while comfort during NHF-1.5 was rather poor (5 ± 1) (*p* < 0.001) (Fig. 1).

**Table 2:** Effects of increasing NHF set flow rate on target physiologic variables

	NHF-0.5	NHF-1	NHF-1.5	ANOVA *p* value
Set flow rate (L/min)	35 (30–35)	65 (60–70)^a^	100 (92–109)^a,b^	< 0.001
PaO_2_/FiO_2_	194 ± 96	211 ± 106	219 ± 118	0.064
SpO_2_ (%)	94 ± 2	95 ± 2	96 ± 2	0.139
Arterial pH	7.40 (7.39–7.43)	7.40 (7.39–7.41)	7.41 (7.40–7.45)	0.105
PaCO_2_ ( mmHg)	36.3 ± 6.4	37.6 ± 5.3	36.2 ± 5.7	0.108
RR (bpm)	20 ± 6^c^	17 ± 5	18 ± 6	0.014
Corrected MV (au/min)	46,440 ± 18,515	48,562 ± 17,781	53,870 ± 17,737	0.068
HR (bpm)	78 ± 16	76 ± 17	77 ± 16	0.391
MAP (mmHg)	76 (62–91)	73 (65–80)	74 (61–89)	0.447

NHF, nasal high flow; 0.5-1-1.5, set flow rate in L/kg PBW/min; PaO_2_/FiO_2_, arterial partial pressure of O_2_/inspired fraction of O_2_ ratio; SpO_2_, peripheral oxygen saturation; PaCO_2_, arterial partial pressure of CO_2_; RR, respiratory rate; HR, heart rate; MAP, mean arterial pressure

^a^Post hoc Dunn’s test versus NHF-0.5 (*p* < 0.05)

^b^Post hoc Dunn’s test versus NHF-1 (*p* < 0.05)

^c^Post hoc Bonferroni test versus NHF-1 (*p* < 0.05)

### Effects of NHF on lung volume and ventilation homogeneity measured by EIT

**Fig. 2:**
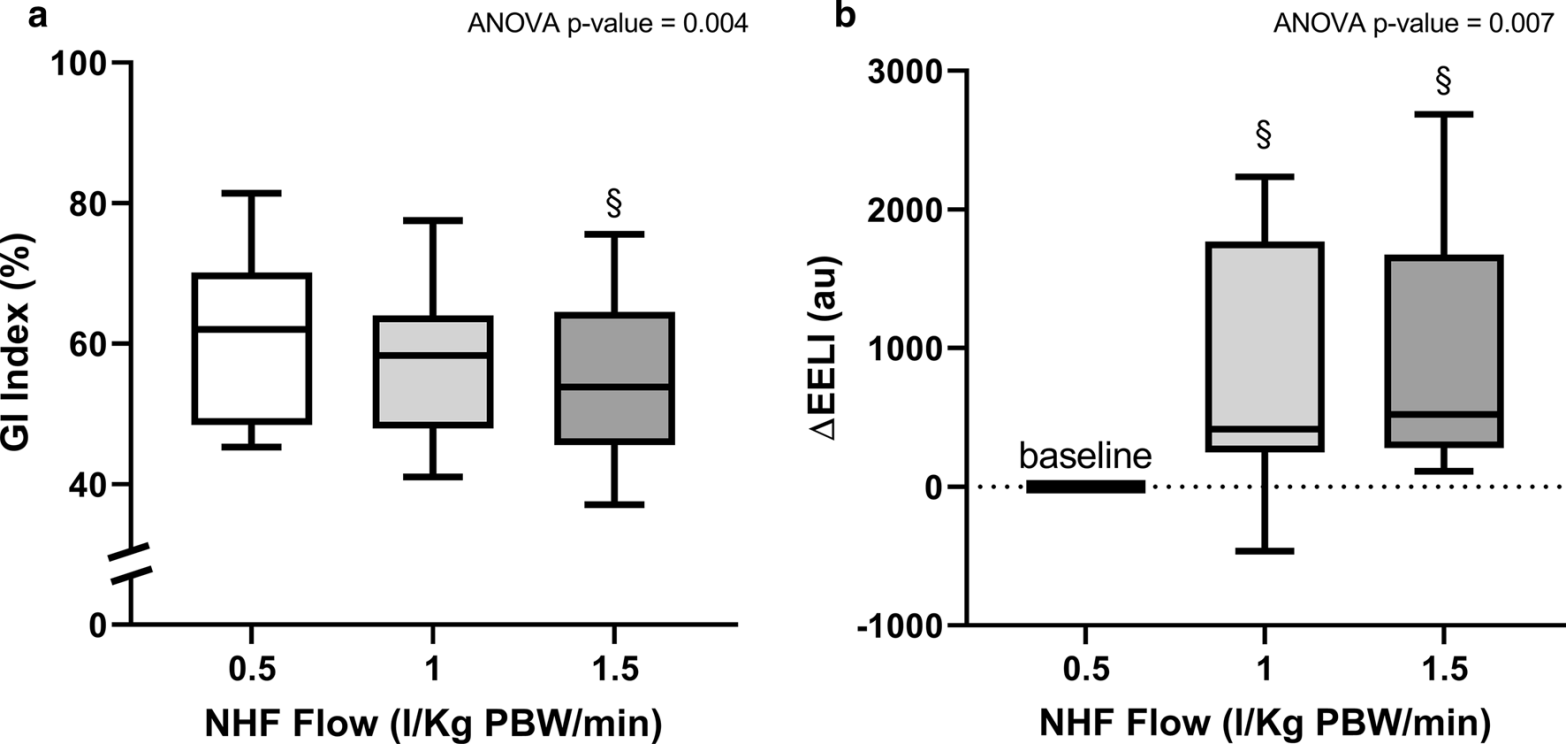
GI index and Global Lung inflation at end-expiration (ΔEELI) during different NHF steps. Global Inhomogeneity Index (GI Index), **a** indicates a more inhomgeneous distribution of ventilation during lower flows. Post hoc correction was performed using Bonferroni test (§*p* < 0.05 vs. NHF-0.5). Lung inflation at end-expiration (ΔEELI), **b** indicates positive pressure effect, and it resulted significantly increased at higher flows. Post hoc correction was performed using Bonferroni test (§*p* < 0.05 vs. NHF-0.5)

**Fig. 3:**
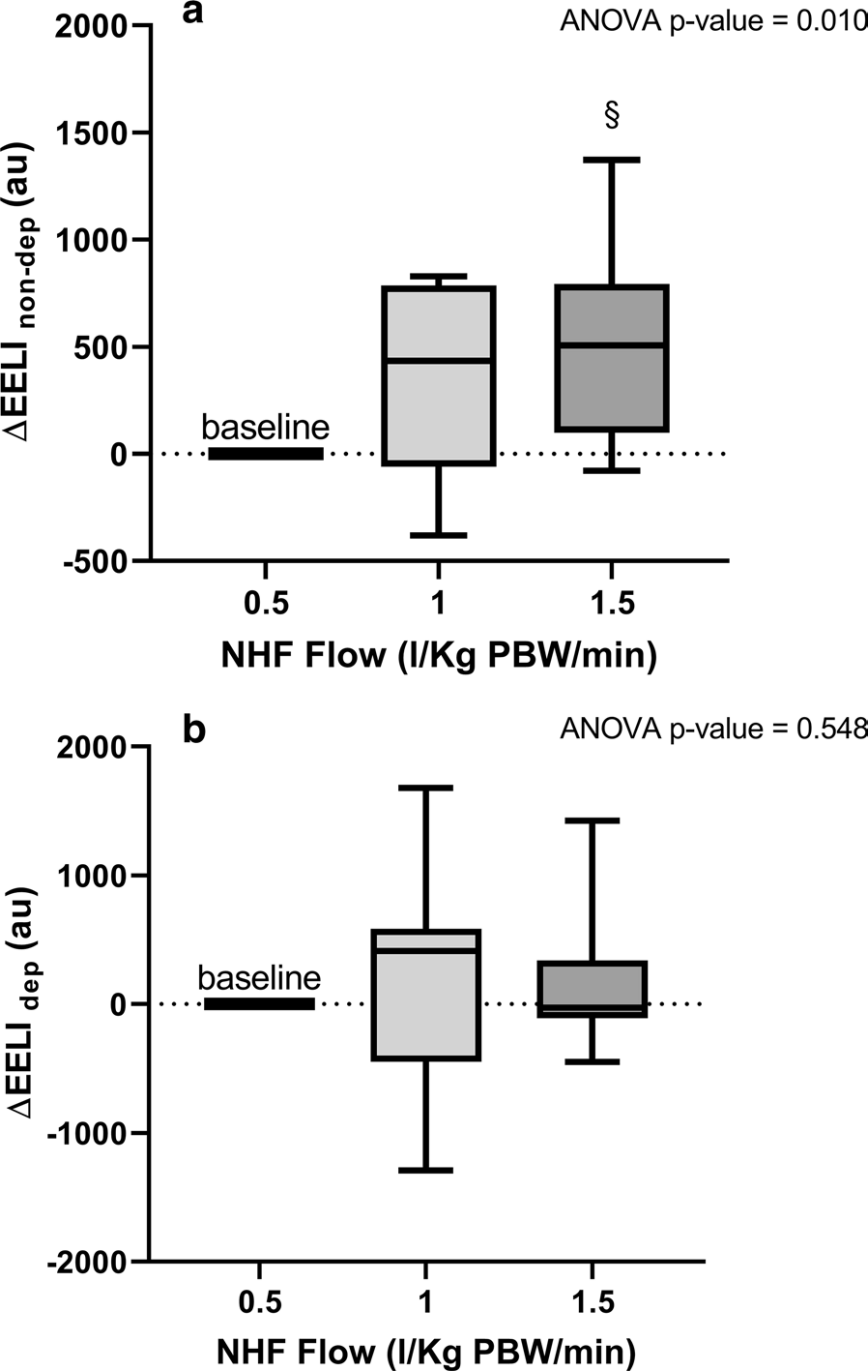
Regional changes in end-expiratory lung impedance (∆EELI_non-dep_, and ∆EELI_dep_) during different NHF steps. The regional changes in end-expiratory lung impedance in non-dependent lung regions, **a** increased during NHF-1 and NHF-1.5 flow: post hoc correction was performed using Bonferroni test (§*p* < 0.05 vs. NHF-0.5). In contrast, end-expiratory lung impedance in dependent lung regions, **b** remained quite constant

GI index (Fig. 2a) decreased significantly switching from lower to higher flows (*p* = 0.004). Lung inflation at end-expiration (ΔEELI) (Fig. 2b) significantly increased at higher flows (*p* = 0.007), indicating positive pressure effect. Regionally, ΔEELI_non-dep_ (Fig. 3a) increased during NHF-1 and NHF-1.5 (*p* = 0.01), while ΔEELI_dep_ (Fig. 3b) remained quite constant (*p* = 0.548).

### Discussion

The present study showed that use of NHF higher than 60 L/min in a selected population of patients with acute hypoxemic respiratory failure is associated with improved physiology in terms of reduced respiratory rate, increased ventilation homogeneity and more pronounced positive end-expiratory pressure effect; self-reported patient comfort, however, is significantly lower in comparison with the flow rates currently used in clinical practice.

A growing body of evidences indicate that in spontaneously breathing subjects at risk of or with established lung injury, NHF is an effective strategy compared to conventional low flow oxygen [19]. The first clinical context for application of NHF was in neonates and infants where it is largely used to decrease risk of re-intubation [20] and manage acute respiratory pathologies such as bronchiolitis [21, 22], asthma and croup [23]. In adults, NHF became extremely popular after publication of the FLORALI study [18] which demonstrated lower intubation and mortality rates in AHRF patients treated with NHF in comparison with noninvasive ventilation and conventional oxygen therapy. Subsequently, other clinical trials expanded indications for NHF in adults to immunocompromised patients and to patients at risk for re-intubation after surgery or in the ICU [24, 25]. However, for the present study, we reasoned that: (1) failure of NHF (i.e., intubation) is still higher than 30% and associated with high mortality rate, especially in the case of delayed timing [14]; (2) in neonates and infants, flow rates indexed per body weight are much higher than those used in the adult population (2–3 vs. 0.5–1 L/kg PBW/min) [26]; (3) Previous physiological studies showed that effects of NHF improve at higher flow rates [17]. Thus, we conceived an exploratory physiological study to assess whether use of NHF at flow rates comparable to the neonatal setting is associated with improved physiology.

Previous studies investigated the physiological effects of humidified NHF which include improved oxygenation, washout of the anatomical dead space, reduction of respiratory rate and inspiratory effort, generation of positive expiratory pressure with increased end-expiratory lung volume [4, 27]. From a physiological point of view, our study showed that NHF delivered at a set flow rate of 1.5 L/kg PBW/min is associated with some benefits. Indeed, reduced respiratory rate, improved ventilation homogeneity and larger increase of end-expiratory lung volume could lead, respectively, to lower work of breathing [28], improved respiratory mechanics [29] and reduced lung strain [4, 30]. All these effects could, in turn, cooperate to reduce the risk of muscular failure and/or of additional lung damage [8]. However, respiratory rate and inhomogeneity index were not lower than during more conventional flow rate of 1 L/kg PBW/min, the clinical impact of decreased inhomogeneity index is still unclear and end-expiratory lung volume increased mostly in non-dependent lung regions, where over distension rather than recruitment usually occurs.

Our results highlighted worsening of patient’s comfort during the NHF 1.5 phase. In previous studies, comfort played a key role in determining the clinical efficacy of NHF. Comfort is significantly higher during NHF in comparison to conventional noninvasive ventilation through face mask [31]. Indeed, NHF can be continuously administered for days versus hours for noninvasive ventilation. Improved comfort after start of NHF is also a predictor of clinical success [32], as if comfort could be seen as a “holistic” index of the improvements generated by NHF (decreased respiratory drive + decreased effort + more comfortable interface + more stable gas exchange + less restraint for patient movement). We previously showed that comfort is influenced by NHF settings, with worse values during high temperature and lower flows in more hypoxemic patients [33]. In this perspective, our result showing significantly poorer comfort during the NHF-1.5 step may be relevant both from a physiological and clinical point of view. Poor comfort might indicate that the patient is facing worsen physiological condition that we weren’t able to measure during the present investigation (e.g., higher expiratory resistance or ineffective humidification). Clinically, poorer comfort might limit tolerance to the device and reduce the time of application during the day, potentially vinifying its physiological benefits. Methods to improve comfort (e.g., modulation of NHF temperature, mild sedation, music intervention, etc.) were not tested in the present study and could be explored to improve tolerance to NHF delivered at very high flow rates.

During the NHF-1 phase, the set flow rates were already relatively high, with 75% of patients receiving ≥ 60 L/min and 25% ≥ 70 L/min. Indeed, such flow rates were associated with reduced respiratory rate and increased end-expiratory lung inflation at an acceptable level of comfort. This finding might suggest that increasing the clinical boundary of NHF set flow rate to 70–80 L/min might be feasible and associated to improved physiology. Moreover, it could be interesting in future studies to assess the effects of intermediate flow rates (e.g., 1.25 L/kg PBW/min).

The clinical judgement about use of new or modified medical devices must take into account both their ability to achieve physiological and therapeutic goals and their feasibility. The burden of collateral effects associated with the new therapy or with necessary adjunctions (e.g., the risks associated with intravenous sedation needed to tolerate the device) should be clearly minor in comparison to the physiological and clinical gain. Our data indicate that NHF delivered at flows higher than 60 L/min is associated with physiological improvements that needs to be weighed against patient’s tolerance and/or risks of strategies needed to improve tolerance.

Despite being the first investigation on AHRF patients treated by NHF at gas rates higher than 60 L/min, this study has limitations that need to be noted. First, it was an exploratory small physiological study and the clinical relevance of our findings need specific validation. Second, we did not performed esophageal pressure monitoring to quantify the inspiratory effort which is the main cause of self-inflicted lung injury and risk of diaphragm trauma. Third, each study phase lasted for a limited amount of time and the physiological effects might evolve along further treatment with NHF. Fourth, despite randomization, we cannot exclude carry over effect due to the small sample size and lack of wash out phase.

## Conclusions

In non-intubated hypoxemic patients, NHF delivered at flow rates higher than 60 L/min provided improvement in physiological effects with the risk of poorer patient’s self-reported comfort. While waiting for larger studies with broader assessment of physiological and clinical outcomes, based on our results, use of NHF delivered at such high rates in clinical practice may deserve close monitoring of the individual patient’s response.

## Data Availability

The datasets used and/or analysed during the current study are available from the corresponding author on reasonable request.

## Abbreviations

AHRF: Acute hypoxemic respiratory failure
BMI: Body mass index
∆EELI: End expiratory lung impedance
∆EELI_dep_: End expiratory lung impedance of dependent region
∆EELI_non-dep_: End expiratory lung impedance of non-dependent region
EIT: Electrical impedance tomography
GI: Global Inhomogeneity Index
HR: Heart rate
ICU: Intensive care unit
MAP: Mean arterial pressure
MV: Corrected minute ventilation
NHF: Nasal high flow
PaCO_2_: Arterial partial pressure of CO_2_
PaO_2_/FiO_2_: Arterial partial pressure of oxygen/fraction of inspired oxygen
PBW: Predicted body weight
PEEP: Positive end-expiratory pressure
P-SILI: Patient self-inflicted lung injury
RR: Respiratory rate
SAPS II: Simplified acute physiology score II
SOFA: Sepsis-related organ failure assessment
SpO_2_: Peripheral oxygen saturation
VNS: Visual numerical scale
